# Communicating climate change and health in the media

**DOI:** 10.1186/s40985-016-0044-1

**Published:** 2017-02-16

**Authors:** Anneliese Depoux, Mathieu Hémono, Sophie Puig-Malet, Romain Pédron, Antoine Flahault

**Affiliations:** 1 Centre Virchow-Villermé, Université Sorbonne Paris Cité, Paris, France; 2 GRIPIC, EA 1498, Université Paris Sorbonne – CELSA, Paris, France; 3 Linkfluence, Paris, France; 4 Institute of Global Health, University of Geneva, Geneva, Switzerland

**Keywords:** Public health, Climate change, Communications, Media, Information sharing, Social media

## Abstract

The translation of science from research to real-world change is a central goal of public health. Communication has an essential role to play in provoking a response to climate change. It must first raise awareness, make people feel involved and ultimately motivate them to take action. The goal of this research is to understand how the information related to this issue is being addressed and disseminated to different audiences—public citizens, politicians and key climate change stakeholders. Initial results show that the scientific voice struggles to globally highlight this issue to a general audience and that messages that address the topic do not meet the challenges, going from a dramatic framing to a basic adaptation framing. Communication experts can help inform scientists and policy makers on how to best share information about climate change in an engaging and motivating way. This study gives an insight about the key role of the media and communications in addressing themes relating to climate change and transmitting information to the public in order to take action.

## Background

It has been proven that human activity has an important impact on climate change [1, 2]. Targeted communication is necessary to engage people to adapt towards a more climate friendly behaviour. Previous literature suggests that a lack of basic knowledge on climate change is one of the largest perceived barriers to taking action [3]. Thus, the framing of the issue by the media has a critical influence on the perception of urgency and willingness to respond [4]. Subsequent studies allow to better understand what communication methods are most effective in inducing behaviour change [3–8]. In particular, two media-impact studies [4, 6] have shown that framing climate change as a public health concern rather than as an environmental issue is one of the elements that would help increase the involvement of the public in engaging with climate change. The goal of this 4CHealth’s^1^ contribution is to understand how information on climate change and health is communicated in two different forms of media, the French newspaper, *Le Monde*, and the social media platform, Twitter.

## Main text

A review of articles in *Le Monde* referring to climate change and health was conducted covering the time period from the release of the first Intergovernmental Panel on Climate Change (IPCC) report in 1990 to the end of the 21st Conference of the Parties (COP21) climate negotiations held in Paris in December 2015. Moreover, during the 6 months prior to the conference, *tweets* referring to the COP21 were collated and the frequency and manner in which the issue was addressed was analysed.

The analysis of *Le Monde*’s articles demonstrated an evolution in the communication surrounding climate change and in its framing. Between 1990 and 2015, 4465 articles mentioned “climate change”; however, only 599 of those articles also mentioned “health” (13,4%) and merely 189 of these linked climate change to its health outcomes (4.2%). Despite the low number of published newspaper articles displaying the health outcomes of climate change, the issue has been gaining prominence in *Le Monde* since 2000, which leads us to believe that the public health frame is becoming more pertinent in climate change reporting. However, the sections in which they appear demonstrate the media’s tendency to frame climate change as an environmental issue [4]. Of the articles, 59.4% are published in the “Planet” section followed by “Ideas”, “Economy” and “International”.

A 2015 study by Maibach et al. highlights the “clear need to better inform on the health threats associated with climate change” [7]. The frequency of studies relating to the health impacts due to climate change in peer-reviewed scientific publications has also increased over the past 25 years [9]. The growing research in this area may indicate the transmission of scientific knowledge to the general public through media channels. This is particularly true of the French media potentially arising from reporting both before and during the COP 21 climate negotiations held in Paris in December 2015. Most articles linking climate change and health in *Le Monde* mentioned extreme climate change events (31%) followed by infectious diseases (23%) and environmental migration related impacts (18%). Malnutrition (10%) and respiratory diseases (8%) and others (10%) remain less represented. However, highlighting the health risks associated with climate change is an ineffective communication method according to Maibach et al. (2008) [5], if it is not accompanied by relevant information regarding potential solutions. Furthermore, “information about the potential health benefits of specific mitigation-related policy actions appears to be particularly compelling” [6]. Even though an increase of the reference to health co-benefits since 2000 is noticeable, we observed that only very few of the analysed articles (16%) provide information about health benefits which relate to regulation policies in favour of climate change.

**Fig. 1:**
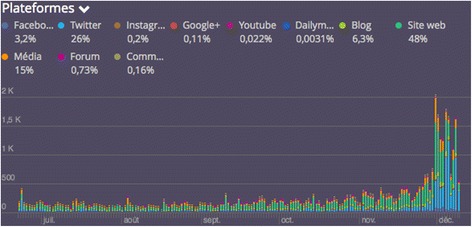
Evolution of the occurrence of the #COP21 related to health from June 15 to December 11, 2015 in the social media: Successful mobilisation of the health sector on Twitter (Data collected by Linkfluence)

On social media, the number of tweets indicating the link between climate change and health, in terms of impacts or co-benefits, has increased during the COP21. A study based on data extracted using Radarly, an extraction tool of social media content (Linkfluence), was conducted to analyse posts including the hashtag “#COP21” from June 15th 2015 (date of the publication of the Lancet Commission on Health and Climate Change [10]) to the end of the COP21 (see Fig. 1). In the run up to the conference, an increased number of health-related posts were monitored. While during the first months, institutional actors expressed a growing interest in the health-related issues of climate change, this topic was endorsed by a diverse range of actors, from the health field (such as Doctors for Climate or the *Ordre des Médecins* in France) to the industrial sector and the civil society. The climate health campaign initiated by the Global Climate Health Alliance, which was very often referenced, as well as the World Health Organization’s call for urgent action to protect health from environmental changes, played an important role in the increase of attention paid to the topic in social media.

## Conclusion

In conclusion, “the lack of constant attention paid to climate change” [3] as well as the lack of efficiency of the message prevent the analysed media from fulfilling its role of provoking a collective response and a change in behaviour. Moreover, information regarding health risks associated to climate change should be framed as a public health threat and supplemented with recommendations and action items provided by experts.

## Abbreviation

IPCC: Intergovernmental Panel on Climate Change

## Footnotes

1 4CHealth is an interdisciplinary project of the Centre Virchow-Villermé for Public Health Paris-Berlin, fostered in partnership with the University of Heidelberg in Germany. It brings together a team of public health researchers, epidemiologists, doctors, political scientists, cognitive behaviour specialists and information and communication scientists.
